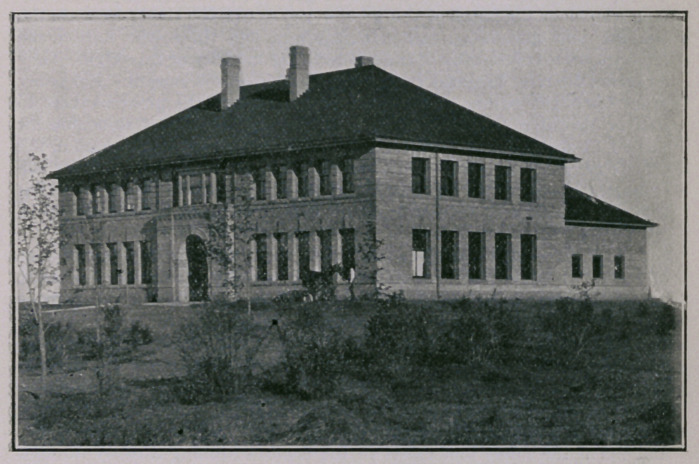# Society Proceedings

**Published:** 1902-10

**Authors:** 


### SOCIETY PROCEEDINGS.

### PENNSYLVANIA STATE BOARD OF VETERINARY MEDICAL EXAMINERS.

The annual meeting of the Board convened at the School of Industrial Art, Broad and Pine Streets, June 16 and 17, 1902, at 9 A. M. each day. Members present: Drs. Jas. W. Sallade, Jacob Helmer, W. H. Ridge, and W. Horace Hoskins; absent, Dr. J. C. McNeil.

The following twenty applicants presented themselves for examination before the Board: W. J. Kirk, Sharon, Pa.; Joseph McClintock, 1720 N. 9th St., Philadelphia, Pa.; Frederick Weitzel, 100 Park Way, W. Allegheny, Pa.; Hyma Feigenbaum, 3224 Butler St., Pittsburg, Pa.; B. Tilghman Woodward, 139 N. 60th St., Philadelphia, Pa; Geo. L. Nicholas, 504 Walnut St., South Bethlehem, Pa.; Robert O. Rothermel, Reading, Pa.; John W. Vansant, Hulmeville, Bucks Co., Pa.; Arthur A. Harmon, Chelmsford, Mass.; H. Clay Campbell, State College, Pa.; Howard Baker, Dudley, Mass.; Summer C. Babson, 3613 Locust St., Philadelphia, Pa.; Guy T. Cole, 122 Benedict Ave., Norwalk, Ohio; Frank U. Fernsler, 621 Chestnut St., Lebanon, Pa.; Samuel Burrows, Vet. Dept., Univ. of Penna., 39th and Woodland Ave., Philadelphia, Pa,; Kenneth E. Paget, 415 Spruce St., Scranton, Pa.; F. C. Bigelow, 1523 N. 55th St., Philadelphia, Pa.; Anthony J. McCloskey, 8707 Shawnee St., Philadelphia, Pa.; John H. Zollinger, 1336 Marshall St., Philadelphia, Pa.; P. J. Purcell, Bradford, Pa.

All of the above-named were granted the license of the Board with the exception of Dr. P. J. Purcell.

A meeting of members of the Board was called to order on June 17, 1902, at 3 P. M., President Sallade in the chair; Dr. McNeil the only absent member. Minutes of the December meeting were read and approved. Secretary reported expenses for the year. President Sallade directed auditing of the report to be made by Dr. Jacob Helmer, who approved the same, and report was ordered filed.

Bills were reported by the Secretary for expenses, clerical, legal, etc., to the amount of one hundred and nine dollars and forty-one cents ($109.41). By the members of the Board for railroad, hotel, etc., to the amount of twenty-one dollars and ninety-four cents ($21.94), and orders were drawn for the respective amounts.

Dr. Helmer moved, and seconded by Dr. Hoskins, that fifteen dollars ($15.00) be donated the School of Industrial Art for the use of their rooms for holding examinations. Carried, and bill paid with others.

The following officers were elected for the ensuing year: President, Dr. Jas. W. Sallade; Secretary-Treasurer, Dr. W. Horace Hoskins.

The Secretary reported several violations of the law at different points in the State, which were referred to the Secretary, with power to take such action as he deemed necessary.

On motion the meeting adjourned.

W. Horace Hoskins,
Secretary.

### AMERICAN VETERINARY MEDICAL ASSOCIATION.

The outcome of the thirty-ninth annual meeting of the Association, held at Minneapolis in September, is yet to be determined. The increasing interest, attendance, and membership of the Northwest veterinarians and of Canada, which has recently come under our wing, are future factors whose value cannot, at this time, be measured. The cordial welcome bestowed at the hands of the Minnesota veterinarians will be remembered with great pleasure, and the few slight unpleasant incidents will be, it is hoped, forgotten in the great good accomplished.

President Winchester's address, which appeared in the September number of the Journal, and which has been commented upon editorially by the Journal, contains much food for reflection, and should be given great consideration in the future work of the organization. His review of the progress of the profession was interesting, entertaining, and instructive. His reference to those whom the Association had lost by death during the preceding year was very touching and a fitting expression of the work done by those deceased members.

It was the first meeting at which the Hawaiian Islands were represented at the American Veterinary Medical Association, and the presentation by Dr. W. T. Monsarrat of a beautiful gavel made of native woods and embellished with horn and silver was gratefully received, and as much appreciated by the members as by the President who accepted it.

The attendance list, which was published in a prior number of the Journal, was the largest at any gathering of the profession in this country, and probably in the world.

The newly-elected members show that the Association is drawing its membership from a wider range of territory than ever before, which should add materially to the strength of the organization from a national point of view. All of those recommended by the Executive Committee were elected to membership.

Among other matters brought before the Executive Committee were the charges against Dr. C. E. Cotton for unprofessional conduct. The Executive Committee, after a thorough examination of the charges and many witnesses, decided that the charges were not well founded, and exonerated Dr. Cotton from the same.

The action of the Association is to be much commended in sustaining the position that had been taken at Atlantic City relative to the expulsion of Dr. Claude D. Morris, and the attempt on the part of those who were willing to defend such action to declare the decision of the Association an illegal one was a very poor defence, in view of the Association's freedom from any legal entangling alliances, and it is to be hoped that the last has been heard of this matter in the National Association.

The committee reports were more complete than in preceding years, one of the best being by Dr. E. B. Ackerman, Chairman of the Committee on Intelligence and Education. This report will be reproduced in a later issue of the Journal. Dr. E. M. Ranck, Chairman of the Committee on Diseases, made an extended report, taking up the subject of anthrax, as suggested at the meeting of 1901. No formal report was made by the Committee on Army Legislation, but Chairman Pearson stated that, in view of the attitude of the present Secretary of War, he deemed it inadvisable to make any special effort at present. No report was received from the Committee on Pharmacopœia, though promises were made that before the next convention its work would be well defined and in the hands of the various members of the committee.

Secretary Stewart, in closing the last year of his official work as Secretary. referred to the great increase in the work of the office, and expressed the desire that this should be considered his last year of service. His well rounded-out career as Secretary was followed by his election to the Presidency, an honor most generously accorded him by the Association. His successor, Dr. John J. Repp, of Iowa, will, no doubt, prove a worthy follower in this important official place, and the members may be congratulated that each strong Secretary has been followed by others developing greater zeal and efficiency in the work.

The expenses of the year were reported upon by Treasurer Lowe, showing total receipts of $2119, and expenses aggregating $1542.17. The Secretary reported a balance on hand of $576.83, and cash in the hands of the Secretary $636.02, making a total of $1212.85, with outstanding bills to the amount of $600.

State Secretary reports were received from the following: Drs. J. C. Norton, Arizona; J. I. Gibson, Iowa; J. S. Butler, Minnesota; C. J. Marshall, Pennsylvania; T. E. Robinson, Rhode Island; and G. R. White, Tennessee. These reports were read by the above secretaries, who were in attendance. Others received and read by title were Drs. J. G. Hill, for Florida; Benj. D. Pierce, for Massachusetts; T. E. Smith, for New Jersey; F. E. Anderson, for Ohio; Benj. McInnes, for South Carolina; S. B. Nelson, for Washington, and L. N. Reefer, for West Virginia. Many of these reports contained valuable suggestions, and furnished much information that should be utilized to a greater extent by the National Association in its work. These reports should also be from a greater number of States, and we hope the incoming officers will be able to secure for the meeting in 1903 a report from every State in the Union and all the provinces represented in the Association.

The Vice-Presidents selected for the ensuing year were Drs. J. G. Rutherford, of Ottawa; W. H. Dalrymple, of Baton Rouge; E. M. Ranck, of Glenolden, Pa.; M. H. Reynolds, of St. Anthony Park, and M. E. Knowles, of Helena, Mont., giving a fair distribution of these officers over the country, and the largest number of votes for any candidate being for Dr. Rutherford (seventy-eight votes), a fitting expression of the complete affiliation of the members within the United States with those of Canada.

There were three candidates for the office of Secretary, Drs. John J. Repp, Ames, Iowa; Tait Butler, of Raleigh, N. C., and W. J. Martin, of Kankakee, Ill., resulting in the election of Dr. John J. Repp.

The office of Treasurer was again bestowed upon the present incumbent.

All other nominations for this office, seemingly, were out of order. Dr. Lowe received his election by acclamation.

A large number of the papers that were on the programme were only read by title. The paper of Dr. W. C. Rayen on "Texas Fever and Its Relations to the Live-stock Interests of Tennessee," which appears in this number of the JOURNAL, was thoroughly discussed by Drs. Salmon, Tait Butler, G. R. White, and Dalrymple.

Dr. S. D. Brimhall's paper on "Hemorrhagic Septicæmia in Cattle" was a very complete one, covering the subject in all its aspects.

The practical paper of Dr. A. H. Baker, on "The Pathogenesis of Equine Pneumonic Emphysema," was the presentation of an every-day subject of much practical importacce.

The presentation of the "Results of Strict Sanitary Regulations in Arizona," by State Veterinarian Dr. J. C. Norton, which appears in this number of the JOURNAL, contains many suggestions, and forcibly demonstrates how much the combined work of the Association depends upon the work of its individual members.

Dr. N. S. Mayo, of Manhattan, Kansas, in presenting the subject of "Poisonous Stock Foods," brought forth a subject that resulted in the expression of widely varying opinions as to where the poison line might be defined in connection with many plants and foods. Drs. A. T. Peters, Tait Butler, and W. H. Dalrymple were active in the discussion of this paper.

The other papers presented that attracted considerable attention were those on "Malarial Fever in the Horse," by Dr. F. Torrance, of Winnipeg, Man.; "Differential Diagnosis between Farcy, Furunculus, and Bursatee," by Dr. C. C. Lyford; "Barrenness in Bovines," by Dr. Charles Schmitt, of Dodgeville, Wis.; "Equisetum Arvense," by Dr. F. A. Rich, of Burlington, Vt.; "The Legitimate Field of the A. V. M. A.," by Dr. Roscoe R. Bell, of New York, and "The Life and Character of Dr. R. S. Huidekoper," by Dr. W. Horace Hoskins, completed the papers that were read before the convention.

The cut accompanying this report illustrates the building in which the surgical clinics were held. The building is a new one erected in connec tion with the Veterinary Department of the State College at St. Anthony Park. It was planned and built under the direction of Dr. M. H. Reynolds, and is most admirably adapted to the clinical work in connection with this institution. Its amphitheatre afforded the best facilities the Association has ever had for holding the surgical clinics.

The surgical operations performed were as follows: cryptorchid castration, Drs. Farmer and McKenzie; radical operation for cribbing, Drs. McNeall and Ward; repulsion of two adjacent molars and filling cavity with gutta-percha, Drs. Moore and Brenton; removal of osteochondrom of a larynx and operation for roaring, Drs. Merillat and Peters; correcting deformity of molars, Drs. Lyman and Foster; radical operation for enlarged bursæ, two cases fetlock, capped hocks, Drs. Lyford and Brimhall; radical operation by owner, with recovery and good results; large thorough-pin; enlarged bursa of lateral extensor; cases illustrating Dr. Lyford's paper on farcy, furunculus, and bursatee; case of furunculus; two cases of farcy; two cases of bursatee.

### NEW YORK STATE VETERINARY MEDICAL SOCIETY.

THE twelfth annual meeting was called to order in the large assembly hall of the Wilson Building, Pierrepont and Fulton Streets, Brooklyn, on Tuesday, September 9th, at 10 A.M., by the President, Professor James Law, with a very favorable attendance considering the unusually stormy weather.

On calling the meeting to order President Law introduced Hon. Richard Young, Park Commissioner of the Boroughs of Kings and Queens, who welcomed the Association to the city in the following well-chosen words:

### ADDRESS OF WELCOME BY HON. MR. YOUNG, PARK COMMISSIONER OF BROOKLYN, N. Y.

MR. CHAIRMAN, LADIES, AND GENTLEMEN OF THE ANCIENT AND HONORABLE PROFESSION—

One would not suppose that your profession was ancient and honorable, looking over the gathering here this morning; it is very remarkable that there is hardly a gray head except my own. I suppose that is because I am not of the profession.

My duty, my pleasant duty, is to welcome you to the city of Brooklyn. We call it the borough of Brooklyn, but I am one of those who still fondly call it the city of Brooklyn, and I am always reluctant to speak of it as the borough of Brooklyn, because it seems to belittle the great city in which you assemble; the fourth city in the Union in population, a city admirable in many things, the city of homes. Perhaps no city in this country could be so properly styled the city of homes as Brooklyn. Here are found the men who move the forces of the greater city across the river; here are the bankers, and merchants, and manufacturers, and those who have made America what it is—many of them here in the old City of Brooklyn. They go across the river to spend their energy and reap the reward. For many years it was the custom of the great merchants and the great bankers to live in Brooklyn, but there came a time when it became fashionable for many of those who had gained great wealth to go over to Manhattan, the other city, where they were convenient to many attractions, the theatre, the opera, the literary field; but there are those who are still so fond of Brooklyn that they cannot and will not leave it. Hence, we have the fourth city in the Union.

I never knew what Brooklyn was until I took charge of the Department of Parks. I found that I could drive thirty miles in one direction without running over the bank, and I could drive fifteen miles in the other direction. We might call this a city built around the parks from my standpoint, the parks being the principal feature in my mind. In this city we have fifty-seven parks and squares and fifty miles of boulevard; and you who are interested in horseflesh can find no more beautiful drives in this country—indeed in any other country—than you will find in the city of Brooklyn. We have a continuous boulevard from Prospect Park to the ocean shore, and one of the finest that exists in the country running directly through Prospect Park, which, indeed, is the finest park in the world, and that I say without boasting but from personal observation. There are no such parks in the world, nor have there been since the world was made, as were laid out by the wise men thirty or forty years ago, who intelligently took up that beautiful site and constructed thereon Prospect Park; and those who are here to-day, after the arduous work is done, should find some way—Dr. Berns can perhaps point a way—of visiting that park and driving down the boulevard to the ocean.

But, gentlemen, it may seem to you singular that the Park Commissioner should welcome you here. Why not the Borough President or the Mayor? The Borough President and the Mayor are away, that is my excuse for being here, and it is a great pleasure to welcome you to our city.

There was a time when the wheel became the fad, and I thought that you might all become mechanics, that your calling had ceased to be. I thought there would be no more use for the horse. When I saw three or four thousand people go by like mad on wheels on Saturday afternoon, I said, "The horse is done for;" and when the automobile came in, many of those who were interested in business that was dependent in a measure upon the horse said, "Well, this is the end of the horse." Sometimes it is, and sometimes the end of the driver, and it needs more attention than any horse I ever met. You never saw a man lying under a horse, but you will find men under automobiles with jacks and screws and all sorts of mechanical contrivances to get the thing to go, and when it goes it goes sometimes too fast and sometimes too slow. But after looking it over and considering it very carefully and watching the driving through the park and studying the condition generally, I have concluded that the horse is going to stay. The cycle company has gone into the hands of the receiver; many of the automobile companies have gone into the hands of the receivers. Your work is beginning. You are beginning to understand conditions; we are all beginning to understand conditions. If anybody had said to me when I was a good deal younger than I am now that a veterinary surgeon was necessary to look after a horse I would have said: "Well, let the horse look after himself. He'll come round all right." But this is no longer the case. The first sign of illness prompts the sending for the doctor. And where a horse is valuable, exceedingly valuable, why not? You are reaching that point where you understand the animal, I suppose, back nearly four thousand years ago; therefore, I speak of your ancient and honorable veterinary profession. The animals were sacred. In some of the countries the animals are so sacred that they are cared for more than human beings. For instance, I was in Turkey two years ago, and in the cities there the dogs are so numerous and are so abundant that you have to pick your steps between them—and you pick your steps cautiously, not for fear of the dog but for fear of the law. You might assault a man and you might or might not be reprimanded, it would depend on the man, whether he were a Mahomedan or a Christian. If he were a Mahomedan, you would be reprimanded; if he were a Christian, you would be commended; but if you were to assault a dog, you would be surely fined. And when you realize that they think the spirit of the departed may be in that animal, you will understand it. In those streets there are thousands and thousands of dogs, and when I say you pick your steps, I mean it literally; and the most scrawny, scurvy, mean-looking curs that I ever saw on the face of the globe are there. A charitable person there is not so apt to give bread to a hungry man, but they go off to the butcher and buy and bring in large pieces of meat of various kinds and distribute it to the impecunious dogs. One of our delights was to throw food from the hotel window to see the gathering of the dogs in that particular section. And that is only a type of the regard paid to animal life. In many of those countries they have more regard for the animal than we do. In China, in Japan, throughout the whole of the Levant and Asia Minor they have the most tender regard for the animal, and a man found abusing an animal would be called to account very quickly. They will load a man in Turkey with a load unbearable—you would think no human being could stand under it—and they will load a horse tenderly. In regard for animals we have not reached them, but we are coming to it. A man overloads an animal or abuses him, and someone will say in the street—you don't know whom, a lady or gentleman—"Here, you stop that," and he knows that he must stop it; if he will not, he is punished. That spirit is growing, and the time is coming when all animals will be as tenderly cared for as they are in those countries we think so far behind us.

I look upon the future of your profession as one of great possibilities. I learn here that many of you are from Cornell. Do you know that is a long step in the right direction. When a young man will set himself apart and give two, or three, or four years to train himself in a profession, that is a long stride in the right direction. It means that you are getting down to the bottom of what you want to do. I am glad that Cornell is doing this work. I am glad that Cornell is doing new work. I am glad to know that you are training young men in forestry and agriculture. When I looked for a man to take charge of the trees of this city and the parks (because all the trees of the city and highways are now in charge of the Park Department), I found a young man just graduated from Cornell, and I set him to work, and he is doing fine work, as his study has fitted him admirably for it. I wanted another man, and there were two applicants. One was a young man from Cornell. I have a letter, received from him this morning, asking me if he can remain another year and finish his course, and then if there will be a chance for him; if not he will come at once. I shall write him to finish his course. I shall write him to remain and finish his course there; there will always be a place for the man who is at the top of his profession whatever it may be; it does not make any difference whether he is in your ranks, whether he is in the medical profession, in law, in literature, in science, or in mechanics; there is always room at the top. Now I say this from a practical business experience in New York of more than thirty-five years.

Year by year there are gathering national associations of manufacturers and all the bodies that formed organizations for the betterment of their conditions and the strengthening of those qualities that go to make up success, and your coming together as other organizations and other professions do throughout the country is doing more for your profession, and for all others, than anything you could conceive of. There was a time when a merchant did not allow his neighbor to know anything of what he was doing. Hence, a man could come to New York and buy from one firm, and another, and another, and when he failed he owed four or five thousand dollars. Now, when he comes, Smith says to his neighbor, "John Jones is in town. How is he? He has bought very largely from me." "Well, better inquire of Brown, he has bought very largely of him." What is the basis of it? We are helping each other. You come together and you discuss matters pertaining to your profession; and every man about you will go home wiser and better able to do his work. You become stronger physically and mentally, and go back stronger in the knowledge of your profession.

I trust, gentlemen, you will have an enjoyable time in this city. I know that you are not going to have much time to run about, and if you do, you will probably run to the other side of the river, you from up the State, because there is not here just as much ast here is over there; but give some attention to this great city, the city of homes, the city of churches, this city where men think and go over there to put into execution.

Mr. Chairman, I thank you for the opportunity of meeting your organization. Ladies and gentlemen, I thank you, for your courtesy in listening to me.

Professor Law replied to the cordial welcome to the "City of Churches" as follows:

RESPONSE BY PRESIDENT JAMES LAW.

On behalf of the New York State Veterinary Medical Society, Mr. Young, it is my pleasant duty and privilege to thank you for your kind and courteous and warm welcome to the city of homes.

True we have somewhat of an old profession, beginning back indefinitely —in one sense beginning very definitely, perhaps, about the Grecian period —but we look upon our profession as, in a sense, dating from only the beginning of the last century. It was at that time that definite systematic education was attempted and veterinary medical colleges incorporated; and as regards the modern veterinary profession, we look upon that rather as our own. This city of homes and churches, Borough of Greater New York, is, in a sense, also young. We have only to go a little further back than our modern veterinary schools of education to find these waters covered by the Indian canoe and the shores dotted with Indian wigwams, and to-day we stop here and are welcomed in a city that is perfectly overwhelming considering the date of its start. We stand in the midst of all this vast material prosperity that comes from our modern physical conditions; we stand here in the city of a million and a quarter, or, taking in New York, I am not sure how many millions, at any rate, in Greater New York we have the prospect of a city that will probably excel any other on the face of the earth, now the metropolitan city of the most progressive country on the earth. And this should be, in a sense, an inspiration to us. The beginnings were small, and necessarily grew slowly, but we, like Brooklyn, have grown fast. We have and are growing healthily. It is for us to help each other, and largely at this convention, where one and all can bring the attention and the energy and the will that will help the advance of the profession as a whole and make them more valuable to the great animal industry. In connection with the veterinary profession, if there is one thing that impressed me more than another, it is an incident in connection with the history of the profession in the United States that is closely allied with Brooklyn. In Brooklyn, away back in 1848, there landed an Englishman. It appeared that he had brought with him a cow which he soon sold. We got not only the cow, but we got lung plague of cattle; and that spread until it extended from New York south and west to all the States of the Mississippi Valley and north to Chicago, to the very borders of the greatest cattle market in the world, and finally infected the whole country. At that time it was estimated that shippers of live cattle to England were losing two millions per annum from the embargo that was placed upon our stock by the British Government. Well, the veterinary profession was called upon to attend to this matter; and, to make a long story short, within a few years the whole infection was wiped out, east and west, north and south, and to-day we stand without a single germ of that fatal lung plague to cattle which was prevalent for nearly forty years and which prevails in the old world very disastrously now. Here is a triumph of our profession that we look upon with pride. I don't wish to depreciate any field of work of the veterinarian, I would appreciate all, but when I say that a man who successfully treats an individual case of illness in one of the valuable animals does well, I say a man who will work for and find a preventive against the possibility of infection or death, does somewhat better, and the profession that can step in and absolutely extinguish the infection from the new world has done best of all.

The President's address, which appears on page 605 of the JOURNAL, was received with great interest and highest appreciation.

The attendance of members and of visitors was as follows: Drs. E. B. Ackerman, Brooklyn; E. F. Alexander, Hoosick Falls; Charles S. Atchison, Brooklyn; Samuel Atchison, Brooklyn; Roscoe R. Bell, Brooklyn; George H. Berns, Brooklyn; A. W. Baker, Brasher Falls; W. L. Baker, Buffalo; A. Barradell, Pawling; H. J. Brotheridge, Brooklyn; H. E.

Bates, South Norwalk, Conn.; C. E. Burchsted, Exeter, N. H.; F. F. Bushnell, Winsted, Conn.; E. M. Casey, Oxford; D. W. Cochrane, New York; Charles Cowie, Ogdensburgh; A. J. Dodin, Morrisania; Thomas H. Doyle, New York; William F. Doyle, Brooklyn; J. F. De Vine, Goshen; Robert W. Ellis, New York; W. E. A. English, Jersey City, N. J.; P. A. Fish, Ithaca; Otto Faust, Poughkeepsie; H. D. Gill, New York; George J. Goubeaud, Brooklyn; G. W. Gilbert, Bayport, L. I.; E. Hanshew, Brooklyn; H. D. Hanson, New York; F. R. Hanson, New York; George S. Hopkins, Ithaca; Wilson Huff, Rome; R. C. Jenks, Ossining; L. B. Judson, Winsted, Conn.; R. E. Jones, New York; E. H. Judkins, New Paltz; M. Kenny, New York; William Henry Kelly, Albany; George A. Knapp, Milbrook; H. W. Kornobis, Brooklyn; James Law, Ithaca; A. M. Leek (veterinary student), Highwood, Conn.; George W. Meyer, New York; R. W. McCully, New York; R. A. McAuslin, Brooklyn; W. T. McCoun, Oyster Bay; V. A. Moore, Ithaca; C. D. Morris, Binghamton; W. B. Moorehouse, Tarrytown; Andrew R. Morris, New York; Edward J. Nesbitt, Poughkeepsie; L. Nicholas, New York; Arthur O'Shea, New York; T. F. O'Dea, Saugerties; R. Perkins, Warsaw; Thomas M. Quinn, Astoria; A. K. Robertson, Brooklyn; J. L. Ronan, Corning; Charles Schroder, Brooklyn; C. E. Shaw, Brooklyn; H. E. Stark, New York; H. D. Stebbins, West Winfield; G. F. Stone, Binghamton; Harry Sutterby, Batavia; T. G. Sherwood, New York; A. G. Tegg, Rochester; A. J. Tuxill, Auburn; E. F. Voorhis, Owego; James W. Walker, Brooklyn; E. Waters, Brooklyn; R. E. Waters, Gravesend; W. J. Wadsworth, Cobleskill; LeRoy Webber, Rochester; R. M. Weightman, Waterville; A. G. Wicks, Schenectady; W. L. Williams, Ithaca; J. L. Wilder, Dunkirk; H. E. Wilson (veterinary student), Brooklyn.

Delegates from the Veterinary Medical Association of New Jersey: Drs. Earnest Buckley, East Orange; J. M. Everitt, Hackettstown; J. B. Hopper, Ridgewood; J. V. Laddey, Arlington; J. Payne Lowe, Passaic; William Herbert Lowe, Paterson; Werner Runge, Newark; S. S. Treadwell, Englewood.

Ladies: Mrs. E. B. Ackerman, Brooklyn; Mrs. Roscoe R. Bell, Brooklyn; Mrs. Charles Cowie, Ogdensburgh; Mrs. H. D. Hanson, New York; Mrs. James Law, Ithaca; Mrs. J. L. Wilder, Dunkirk; Mrs. W. L. Williams, Ithaca.

Other visitors: A. R. Davidson, Brooklyn; John A. Dunn, Brooklyn; T. F. Krey, New York; Hon. Richard Young, Brooklyn.

The following veterinarians, whose applications were favorably reported upon by the Executive Committee, were elected to membership: Drs. Harry W. Kornobis, Brooklyn; R. C. Jenks, Ossining; Richard M. Weightman, Waterville; Ernest F. Alexander, Hoosick Falls; Otto Faust, Poughkeepsie; H. J. Brotheridge, Brooklyn; George A. Knapp, Milbrook; Edward J. Nesbitt, Poughkeepsie; Robert J. McAuslin, Brooklyn; George W. Meyer, New York; C. R. Perkins, Warsaw; Thomas M. Quinn, Astoria; E. H. Judkins, New Paltz; Charles Schroder, Brooklyn; Andrew R. Morris, New York; James W. Walker, Brooklyn; D. W. Cochrane,

New York; William J. McKinney, Brooklyn; Le Roy Webber, Rochester; Herbert Sheldon Sackett, Brooklyn; Frank Hunt, Jamestown; C. E. Shaw, Brooklyn; A. G. Tegg, Rochester; R. E. Waters, Gravesend.

The reports of the several committees were then received. Outside of the Committee of Arrangements little active work had been done.

The Executive Committee reported upon the charges against Secretary Claude D. Morris as follows:

1. That Dr. Morris was indiscreet in using this Society's paper in writing a personal letter to the Secretary of War.

2. That Dr. Morris was indiscreet in stating in said letter that "the profession at large is not at heart for the enactment of this measure."

This committee feels that while Dr. Morris failed to exercise due discretion, they do not think that his purposes were such as to warrant the imposition of any penalty.

After the report was received a motion was offered by Dr. Bell that the defendant be tried in open session, which was carried. The defendant asked that he be tried in executive session. All except members were asked to withdraw. After due consideration the report of the Executive Committee was adopted.

The following papers were then read and discussed: Dr. Robert W. Ellis, "Veterinary Dentistry;"[1] "The Etiology of Shoe-boil,"[1] by Dr. George J. Goubeaud; "Retained Placenta," by Dr. W. L. Williams, page 620; "Syrup as a Food for Horses,"[1] by Dr. George H. Berns, of Brooklyn. "Mallein as a Diagnostic Agent" was presented by Dr. H. D. Gill.

Other papers were submitted by title as follows: "Interstitial Hepatitis of Swine," by Dr. V. A. Moore; "Veterinary Dentistry," by Dr. Childs; "Laryngeal Paralysis or So-called Cerebro-spinal Meningitis," by Dr. Gill, and "The Diagnosis of Anthrax," by Dr. Moore, in order that the subject of the enforcement of our veterinary laws introduced by Dr. Kelly might be considered.

After a thorough discussion of the need of a more rigid enforcement of the laws regulating the practice of veterinary medicine in New York State, the following resolutions were adopted:

WHEREAS, The Pennsylvania State Veterinary Medical Society has seen fit to criticise the action of this Society as to its method in dealing with one of its members; and

WHEREAS, This Society feels that it is perfectly capable of administering its own By-laws in its own way, without the advice, sanction, or interference of those from without its membership; therefore, be it

*Resolved*, That this Society regards as indiscreet, discourteous, and gratuitous the resolutions passed at the last annual meeting of the Pennsylvania State Ve nary Medical Association.

*Resolved*, That the New York State Veterinary Medical Society recognizes in the death of Rudolf Virchow, pathologist, scientist, and statesman, an irreparable loss to medical science and humanity—a man of universal

[1] This will appear in a later number of the JOURNAL.

genius and a benefactor of mankind, who, starting early in life in advance of his line, maintained that standard throughout his long career.

In recognition of these qualities we manifest the same by silent vote in rising.

PROPOSED AMENDMENTS TO THE CONSTITUTION.

We the undersigned members of the New York State Veterinary Medical Society offer the following amendments to Article V. of our Constitution:

"To strike out the word 'fifteen' and substitute 'twenty-five' in the fourth line."

"To strike out the word 'two' and substitute 'two or three' in the sixth line."

We also beg to offer the addition of a new article to the Constitution, to be known as Article No. VII., to read as follows:

"That a standing committee of three members be appointed as a Committee on Resolutions."

George H. Berns,
W. L. Baker.

The next meeting, by vote, was decided in favor of Ithaca, N. Y.

The surgical clinic, which was held in the hospital of Dr. George H. Berns, 74 Adams Street, included the presentation of the following cases:

1. Practical demonstration of the use of the ophthalmoscope for the diagnosis of obscure lesions of the eye. By Dr. George G. Van Mater, of Brooklyn.

2. Enormous enlargement and induration of both parotid glands in a gray horse (probably melanosis). By Dr. George H. Berns, of Brooklyn.

3. Lacerated wound of the inferior abdominal region, with protrusion of the intestines and discharge of alimentary matter through wound. By Dr. George H. Berns, of Brooklyn.

4. Dropping of stifle, following azoturia, three months' standing. By Dr. George H. Berns, of Brooklyn.

5. Gelatinous degeneration of the pastern, following plantar neurotomy. By Dr. George H. Berns, of Brooklyn.

6. Large cystic tumors of the poll. By Dr. George H. Berns, of Brooklyn.

7. Periostitis of pedal bones, both front feet. By Dr. George H. Berns, of Brooklyn.

8. Peculiar action of the stifle and relaxation of the tendo Achilles. By Dr. William J. McKinney, of Brooklyn.

9. Aggravated stringhalt of both hind legs. By Dr. Roscoe R. Bell, of Brooklyn.

10. Suspected case of farcy. By Dr. George H. Berns, of Brooklyn.

11. A pair of truck horses doing daily work fed on molasses food for eighteen months (property of Arbuckle Bros.).

12. Aggravated case of stringhalt. By Dr. J. L. Robertson, New York.

The following operations were performed:

1. Tenotomy of deep flexors, one front and both hind legs. By Dr. G. A. Stone, Binghamton.

2. Ovariotomy of mare through vagina (with privilege to all of passing hand through wound and feeling the parts). By Dr. W. L. Williams, of Ithaca.

3. Extirpation of membrana nictitans. By Dr. George H. Berns, of Brooklyn.

4. Castration of stallion, standing (time, sixty seconds). By Dr. R. E. Waters, of Gravesend.

5. Tenotomy, deep flexors both hind legs, donkey. By Dr. George H. Berns, of Brooklyn.

6. Tenotomy of lateral extensor of the phalanges (stringhalt operation). By Dr. C. E. Shaw, of Brooklyn.

7. Extirpation of lateral cartilage for the radical cure of quittor. By Dr. W. F. Doyle, of Brooklyn.

8. Demonstration of the use of a new mechanical tooth float. By Dr. Robert W. Ellis, of New York.

9. Demonstration of the use of stocks and operating-tables. By Drs. Joseph R. Hodgson, of Brooklyn, and W. J. McGee, of New York.

After the clinic was held a special trolley trip was made to Coney Island, which ended a very successful and enjoyable meeting.

### WISCONSIN SOCIETY OF VETERINARY GRADUATES.

THE State Society of Veterinary Graduates met at the Kirby House, Milwaukee, September 11, 1902, at 7.30 P. M. The meeting was called to order by the President, and the following members answered to roll-call: S. Beattie, H. P. Clute, C. M. Crane, C. E. Evans, H. F. Eckert, R. S. Heer, J. T. Hernsheim, E. L. Morgonworth, J. T. Roub, and S. S. Snyder. Visitors present: Drs. W. T. Schwiesum and T. A. Schneekloth. The minutes of the last meeting were read and approved.

The President re-appointed Dr. H. P. Clute on Committee of Legislation, as his term had expired.

The Revisionary Committee came under discussion and was dismissed by the Chair.

Dr. Clute, on behalf of Mrs. Stater and Ormond, extended thanks to the Society for their kind remembrance of the late Dr. C. H. Ormond.

Dr. Eckert reported some very interesting experiences with rabies, in which there were six persons infected, all taking the Pasteur treatment successfully.

Applicants for membership were taken, and the following gentlemen were received: Dr. W. T. Schwiesum, Ripon, Wis., and Dr. T. A. Schneekloth, Lodi, Wis. The Censors reported favorably, and the gentlemen were unanimously elected.

On motion, the Secretary was instructed to call a meeting of the Legislative Committee at Madison in October to frame a bill for the next Legislature, the railway expenses of this committee to be paid by the Society.

On motion, the Society adjourned to meet in Madison, subject to the call of the President and Secretary.

S. BEATTIE,
Secretary.

### PASSAIC COUNTY VETERINARY MEDICAL ASSOCIATION

The regular monthly meeting of the Association was held at Dr. William Herbert Lowe's office, corner of Paterson and Van Houten Streets, Paterson, N. J., on Tuesday evening, September 16, 1902, at 8 o'clock, with President Lowe in the chair.

On roll call the following members answered to their names: Drs. Wm. J. Reagan, John H. De Graw, William C. Ferguson, T. J. Cooper, Alexander Machan, William H. H. Doty, W. H. Lowe, Jr., M. A. Pierce, and William Herbert Lowe, of Paterson; Dr. Anthony P. Lubach, of Passaic.

The minutes of the last meeting were read by the Secretary, and on motion of Dr. Cooper were duly approved.

Upon request of members, President Lowe, as delegate to the American Veterinary Medical Association, gave at some length a report of the great veterinary convention held at Minneapolis, Minn., September 2, 3, 4, and 5, 1902.

The President also gave a report of the meeting of the New York State Veterinary Medical Society, held in Brooklyn, N. Y., September 9, 10, 1902.

Dr. Ferguson, *Chairman*, Drs. Doty and Reagan Committee on Constitution, By-Laws, and Code of Ethics, presented a report that was concise and comprehensive. Upon motion of Dr. Pierce the report was received and its recommendations taken up section by section. With slight amendments the Constitution, By-laws, and Code of Ethics were adopted as recommended by the Committee. Upon motion of Dr. Cooper it was ordered that the Constitution, By-laws, and Code of Ethics be printed.

Upon motion of Dr. Lowe, Jr., the bill of the Guardian Printing and Publishing Company for stationery and printing, amounting to $9.75, was ordered paid.

Several matters of local interest to the profession were discussed, and the meeting adjourned at 11 P.M.

A. Machan,
Secretary.

### PENNSYLVANIA STATE VETERINARY MEDICAL ASSOCIATION.

The semi-annual meeting of the Association was held at Reading on September 16, 1902.

The Entertainment Committee treated the members to a pleasant drive over the Neversink Mountain before the opening of the meeting. This is one of the pleasantest drives in the State, and was thoroughly enjoyed by the thirty members who were in attendance.

The meeting was called to order in the Board of Trade Room, No. 25 North Sixth Street, at 12.30, by President Rhoads.

The reading of the minutes of the annual meeting was suspended.

The President delivered his semi-annual address, which was both interesting and instructive.

A recess of fifteen minutes was taken for the collection of dues.

Dr. Pearson, as the Chairman of the Board of Trustees, was present, and said that the committee had no report to make at that time.

Recording Secretary E M. Ranck read the reports for the County Secretaries. These reports are filed with the Secretary.

Correspondence was read from Drs. Butterfield, Ridge, St. Clair, Schneider, Waugh, Bethune, and Jones. Their letters showed much interest on the subject of good roads, and were thoroughly discussed by the members present. It was the sense of the meeting that our Association and veterinarians throughout the State should use their influence to obtain the assistance of the State in building better roads. The Road Drivers' Association of Pennsylvania is already doing good work in this line, and we should assist them in every way possible.

Under reports of delegates, Dr. Hurley, of Dover, made a report for New Jersey, and stated that the profession in his State was in a prosperous condition. He also spoke of the improvement in their county roads since the State had become interested in the matter.

Dr. Pearson made a report for the Keystone Veterinary Medical Society, of Philadelphia. The work done by this Society in reference to the milk-supply for hospitals and public institutions revealed the fact that the conditions for supplying good milk were bad, and that no precautions were being taken to improve this state of affairs. He said that his Society was endeavoring to interest the managers of these institutions in the subject of requiring some system of milk-inspection.

Dr. Noack, of Reading, reported for the Schuylkill Valley Society. This Association is in a prosperous condition, but has had some trouble in dealing with illegal practitioners in this section of the State. Dr. Noack is very much in favor of a Re-registration Act whereby the obsolete and chaotic registration list of the present time can be dispensed with. This seems to be the only way to free the list of the names of men who have ceased to be identified with our profession. He reported that the Association was meeting with some success in the subject of milk-inspection.

Dr. John E. Spindler, of Pittsburg, sent a written report for the Committee on Sanitary Science and Police. His report was read and filed with the Secretary. Dr. M. J. Christman, of Sugar Grove, also made a report as a member of this committee.

A report from the Committee on Legislation was next called for. Dr. George Jobson, of Franklin, made a verbal report. He spoke of several cases where poor people had been bitten by rabid dogs in his section of the State. The Pasteur treatment is the only recognized treatment for this trouble. This treatment costs $150, and poor people cannot afford to take it. He thought in cases of this kind that the State should pay for the treatment. At present, if stock are bitten by rabid dogs and contract hydrophobia the State will reimburse the owner for his losses out of the dog tax money. He thought the State should provide some such plan to assist poor people who have been bitten.

Dr. Jobson also spoke of the necessity of a re-registration for veterinarians, and thought that this matter should be put in the hands of the Board of Veterinary Medical Examiners, and not left with the prothonotaries of the different counties, as it is at present.

Dr. Bowers spoke of the necessity of working up interest on the subject of good roads; that the roads in our State for a portion of the year were nearly impassable, and were a disgrace to the State.

The meeting was adjourned for lunch at 1.30 P.M. It was called to order again at 3 P.M. by President Rhoads.

A report from the Committee on Army Legislation was called for. Dr. W. Horace Hoskins, as Chairman of this committee, spoke of the great loss sustained in the death of Dr. Huidekoper. Very little has been done by the committee since his death. He also spoke of the treachery of Dr. Claude D. Morris. The victory he obtained in defeating the Army bill is enjoyed only by himself, while 8000 other veterinarians in America are disgusted with the course taken by him, and consider him nothing but a traitor. Dr. Hoskins thought that the efforts made by our profession for recognition in the army has done a great deal of good. Veterinarians in the army have noticed a vast improvement. They are consulted more and have a much better social standing. These things will assist a great deal in getting the required recognition in time.

Dr. Pearson made a few remarks as a member of this committee, and urged the necessity of agitating the subject of army recognition.

The Committee on Milk Inspection, of which Dr. Seidel, of Bryn Mawr, was the only member present, made no report.

No report was made either by the Committee on Intelligence and Education or the Committee on Animal Husbandry.

President Rhoads appointed as temporary members for the Board of Trustees Dr. James B. Rayner and Dr. A. W. Weir, who were to serve in the absence of Dr. Thomas B. Rayner and Dr. N. Rectenwald.

Under the subject of new business President Rhoads described in detail the efforts being made by the Road Drivers' Association of Pennsylvania in obtaining necessary legislation and State assistance in making good roads. The circular letters sent to veterinarians in the State on this subject by President Rhoads had been recognized by many who were enthusiastic in this matter and willing to assist in every possible way. The President said one good way to interest people in the subject was to organize Road Drivers' Associations wherever possible. He suggested that our County Secretaries should take up this question and try to work up interest and report to our annual meeting the success or failures which have met their efforts. The subject was well discussed by several members. Dr. E. M. Ranck was in favor of framing suitable resolutions on this subject, but Dr. Hoskins thought best to postpone further efforts in this line until after the evening session, as he understood an evening session would be devoted to this subject.

Dr. Ranck brought up the subject of the advisability of veterinarians keeping a black-list. In some ways he thought such a list might be useful to our Association. Dr. Noack thought such a list would be worthless, because a person who is bad pay with one veterinarian may be and often is very good pay for some other practitioner.

Dr. Hoskins was in favor of discussing this subject thoroughly, but thought action in the matter should be taken only after due consideration and with much precaution, for the reason that our Association is an incorporated body and is liable to prosecution. Only about 12 per cent. of the veterinarians in the State are members of the Association. A black-list could do very little good, and might do a great deal of damage.

Dr. Jobson thought that this subject would be putting too much work on the officers of our Association. He moved that the subject be laid on the table. The motion was seconded and carried unanimously.

Under the subject of new business Dr. Pearson brought up the question of re-registration, and discussed fully the necessity for such a measure. Under the old law about 1700 men registered as veterinarians. Many of these men were policemen, stablemen, etc., who had no right to practise. Of this number only about one-third are regular practising veterinarians.

The Pharmacy Board requires a re-registration every two years. He thought that if veterinarians had similar requirements we could soon get a clean list, and that our present law is now so well founded that it would be comparatively easy to get the necessary amendment passed.

Dr. Sallade urged the necessity of making some definite move in reference to this subject at the meeting. He said the agitation of this subject was started by the Board of Veterinary Medical Examiners. The only assistance this board has ever received was from the State Society. The board is handicapped at present by having nothing but the old obsolete list, which was prepared in many cases by thoughtless or dishonest prothonotaries. The board is also handicapped in not having the necessary funds with which to carry on its work. The money obtained by a re-registration would put the board in good financial standing. They would then have money provided to carry on investigations and prosecutions, if necessary, of illegal practitioners. With the revised list and the required amount of money the work would be comparatively easy.

Dr. Hoskins spoke of the difficulty recently encountered in prosecuting a man by the name of Brensinger, in York County. He was illegally registered several years ago, but in order to prove this it would take a long legal contest and require considerable money to carry on such a suit. In Lancaster County sixty-eight men were registered, while there were only sixteen qualified men in the county.

President Rhoads thought best, in order to economize time, to refer this matter to a Resolution Committee.

The Secretary received a letter from Mrs. Davis, widow of the late Dr. Davis, soliciting assistance from the Association in paying off the mortgage on her house and lot. Dr. Pearson moved that the Secretary be instructed to receive and transmit to her such sums as the members might wish to contribute.

Dr. Pearson suggested that the Committee on Resolutions meet with the Board of Trustees. They adjourned to the committee-room for the transaction of such business as might be brought before them.

The next order of business was the reading of papers. In the absence of Dr. W. S. Phillips, of Reading, his paper on the subject of "Unusual Symptoms in a Three-year-old Filly" was read by the Secretary.

Dr. C. J. Marshall, of Philadelphia, read a paper entitled "A Day's Work." This brought forth an interesting discussion on the subject of the causes and treatment of laminitis. Dr. Ranck described how he had seen horses with laminitis treated at the sales stables in New Orleans. The plan there was to cast the patient and keep him off his feet as much as is possible. The horse was kept down two or three days and turned frequently. He also spoke of a custom in some places of treating this trouble by giving large doses of alum as a drench.

Dr. Hurley, of New Jersey, believed in using cold-packs or soaking the feet in cold water in the first stages, followed by hot water after the acute symptoms have disappeared. He spoke very highly of pads which put pressure on the bottom of the feet, to prevent or cure the condition of drop sole. He considered it bad treatment to cast a horse in this condition, but would allow a horse to do as he pleased about standing.

The report of the Board of Trustees was read by Dr. Pearson. The board reported favorably on the three following applications for membership: Dr. I. W. Zellers, W. G. Huyett, and U. S. G. Bieber, who were unanimously elected.

Dr. Pearson was called to explain the State live-stock sanitary laws of the different States. Hardly any two States have the same system of dealing with this subject. In some States this power is vested in the Governor, in others in different kinds of sanitary boards. He showed that where a competent veterinarian has charge of the work, or is closely identified with the work, much better results are obtained. He cited as an illustration the work done in Arizona, where a competent veterinarian has charge of the work, as against Texas, where the authority is vested in practical laymen. The climatic conditions are about the same. In Arizona, Texas fever is seldom seen. Tuberculosis and glanders are very rare, and all other contagious or infectious diseases have been reduced to the minimum ; while in Texas these diseases are prevalent to an alarming extent. This condition has been recognized by other States, and rigid quarantine measures have been passed against receiving cattle from Texas. With proper veterinary inspectors and directors this condition of affairs could be wiped out, which would place Texas in the front ranks of stock raising.

Pennsylvania was the first State to have a laboratory in connection with its State Board. Other States have followed our example. Massachusetts has spent the most money of any State in the Union in trying to exterminate tuberculosis. Illinois had passed some of the most arbitrary laws on this subject. They thought best to test all the cattle in the State, and pay for them according to the progress of the disease. A cow with tuberculosis that had existed five years, so much ; one two years, so much, and so on down to the starting point of the disease. This idea was originated by five laymen who are authorized to look after the contagious diseases in that State.

Under all these regulations and different systems the confidence in the work is governed principally by the accuracy of carrying out the requirements.

He compared the value of live-stock in the country with other branches of industry. The output of the gold and silver mines of the United States combined would buy but one-fourth of the milk product. The product of the poultry yards was nearly nearly four times the value of the combined output of all our gold mines. An industry of such vast proportions should be guarded with the utmost possible skill and precaution.

Dr. Hoskins read the report of the Committee on Resolutions. These were adopted *seriatim* except the last one, which was laid on the table. (Resolutions will appear in November number).

Dr. Hoskins was then called to report on the proceedings of the American Veterinary Medical Association, which met in Minneapolis in September. He said, in part, that this was one of the best meetings our National Association has ever held. Representatives were in attendance from all sections of North America and even from the Philippine Islands. The Entertainment Committee had made complete arrangements and executed its work perfectly. Every member and visitor received genuine Western hospitality. The ladies in attendance were highly pleased with the attention shown them. Among the points of interest visited by them might be mentioned a trip to the State Fair, State Farm, a ride on Lake Minnetonka, the Falls of Minnehaha, a visit to one of the largest flour mills in the world, and numerous trolley rides. Altogether this was a record breaker in every respect. He hopes that the ladies will continue to accompany their husbands to the meetings of our National Association.

He also spoke highly of the honors received by our Pennsylvania men at this meeting. The new Secretary , Dr. John J. Repp, of Ames, Iowa, was formerly a Pennsylvania man. Dr. E. M. Ranck, of Philadelphia, was chosen as one of the Vice-Presidents. It was pleasant to hear our State Live-stock Sanitary Board spoken of so highly from so many different sources. He concluded his remarks by urging the members to attend our national meeting, and spoke of the different cities that had extended invitations for the next annual meeting. Among the cities most highly spoken of by him was Ottawa, Canada. He asked for an expression of this Association as to the city most suitable for the next meeting of the American Veterinary Medical Association.

Dr. Pearson spoke of the hearty manner in which Dr. Rutherford, of Ottawa, had extended an invitation at the Minneapolis meeting to visit his city in 1903. It was the sense of the meeting that Ottawa should be chosen as the next meeting-place for the National Association. The Secretary was instructed to make this fact known to the Executive Committee of the American Veterinary Medical Association.

Dr. Pearson said that the Legislature would be in session before our annual meeting, and he moved that our Legislative Committee and the Executive Committee be given power to act in whatever way seemed advisable in reference to new legislation. The motion was adopted.

The meeting was regularly adjourned to meet in Philadelphia on the first Tuesday after the first Monday in March, 1903.

An evening session was held in the Opera House. This session was devoted to the subject of good roads. Mr. H. B. Fullerton, of New York City, gave a very interesting address on this subject, which he illustrated by stereopticon views of roads throughout the world. He understood his subject perfectly, and showed plainly the advantages to any country of having good, solid roads.

C. J. MARSHALL,
Recording Secretary.

COUNTY SECRETARIES 1902–03.

Adams, M. Moriarity, Gettysburg; Allegheny, Jas. A. Waugh, Allegheny; Beaver, Jas. B. Buckham, Beaver Falls; Berks, U. S. G. Bieber, Kutztown; Bradford, J. C. Kingsland, Canton; Bucks, H. W. Turner, Lahaska; Cambria, W. B. Prothero, Johnstown; Carbon, Geo. K. Swank, East Mauch Chunk; Center, W. W. Fry, Pine Grove Mills; Chester, Jas. B. Rayner, West Chester; Clinton, C. R. Good, Lockhaven; Crawford, W. M. Wilson, Hartstown; Cumberland, S. P. Bishop, Carlisle; Dauphin, J. H. Oyler, Harrisburg; Delaware, B. M. Underhill, Media; Erie, John Bryce, Erie; Fayette, Geo. Magee, Uniontown; Franklin, C. M. Strickler, Greencastle; Indiana, D. A. Gorman, Hortons; Jefferson, J. G. Bethune, Punxsutawney; Lackawanna, Jacob Helmer, Scranton; Lancaster, E. W. Newcomer, Mt. Joy; Lawrence, E. C. Porter, New Castle; Lebanon, M. J. Collins, Myerstown; Lehigh, M. W. Keck, Slatington; Luzerne, Edwin Hogg, Wilkesbarre; Lycoming, C. W. Holley, Muncey; McKean, C. S. McKenna, Kane; Mercer, A. W. Weir, Greenville; Montgomery, E. W. Powell, Bryn Mawr; Northampton, A. W. Radley, Bethlehem; Northumberland, Harry T. McNeal, Milton; Philadelphia, Geo. S. Fuller, Philadelphia; Schuylkill, J. W. Sallade, Schuylkill Haven; Snyder, A. R. Potteiger, Selinsgrove; Susquehanna, J. F. Butterfield, Montrose; Tioga, S. Nichols, Wellsboro; Venango; Geo. B. Jobson, Franklin; Warren, W. P. Bagnall, Warren; Washington, W. J. Waugh, Washington; York, R. T. Mumma, Hanover.

DELEGATES, 1902–03.

To Schuylkill Valley Veterinary Medical Association: J. C. Foelker, Allentown; A. W. Radley, Bethlehem; Edwin Hogg, Wilkesbarre.

To Lehigh Valley Veterinary Medical Association: J. H. Oyler, Harrisburg; M. Moriarity, Gettysburg; S. P. Bishop, Carlisle.

---

Lyman & Lyman, veterinarians, 332 Newbury St., Boston, Massachusetts, is the new firm just established, composed of Dr. Charles P. Lyman (late Dean of the Veterinary School of Harvard University, and of Lyman & Osgood) and his son, Dr. Richard P. Lyman, recently in practice at Hartford, Conn. A complete veterinary hospital has been splendidly fitted up, with accommodations for horses, dogs, and cats. The circular announcement of the new firm was an innovation that may not bear the best of fruit in the way of advertising.

## Figures and Tables

**Figure f1:**